# Leveraging HIV Program and Civil Society to Accelerate COVID-19 Vaccine Uptake, Zambia

**DOI:** 10.3201/eid2813.220743

**Published:** 2022-12

**Authors:** Patricia Bobo, Jonas Z. Hines, Roma Chilengi, Andrew F. Auld, Simon G. Agolory, Andrew Silumesii, John Nkengasong

**Affiliations:** Ministry of Health, Lusaka, Zambia (P. Bobo, A. Silumesii);; US Centers for Disease Control and Prevention, Lusaka (J.Z. Hines, S.G. Agolory);; Republic of Zambia State House, Lusaka (R. Chilengi);; The Global Fund, Geneva, Switzerland (A.F. Auld);; Africa Center for Disease Control, Addis Ababa, Ethiopia (J. Nkengasong)

**Keywords:** COVID-19, 2019 novel coronavirus disease, coronavirus disease, severe acute respiratory syndrome coronavirus 2, SARS-CoV-2, viruses, respiratory infections, zoonoses, COVID-19 vaccines, HIV, Zambia

## Abstract

To accelerate COVID-19 vaccination delivery, Zambia integrated COVID-19 vaccination into HIV treatment centers and used World AIDS Day 2021 to launch a national vaccination campaign. This campaign was associated with significantly increased vaccinations, demonstrating that HIV programs can be leveraged to increase COVID-19 vaccine uptake.

COVID-19 vaccine scale-up in Africa, the continent with the lowest vaccine coverage, is a current regional and global priority. As of May 1, 2022, only 17% of persons in Africa had been fully vaccinated (*1*). Initial vaccination campaigns in Africa were hampered by lower-than-forecasted vaccine donations (*2*). However, through efforts from multiple stakeholders, the vaccine supply to countries in Africa increased in the latter half of 2021. However, with increasing vaccine availability, new challenges became apparent, including the difficulty for under-resourced health systems with relatively low healthcare worker–to–population ratios to implement COVID-19 vaccination services, as well as difficulties reaching populations unaccustomed to adult immunization programs and vaccine misperceptions and misinformation. Facing these challenges, in August 2021, the government of Zambia worked with stakeholders to leverage its national HIV program (which has been supported by >$5 billion in funding in the previous 20 years) to enhance its COVID-19 vaccine campaign.

Zambia integrated COVID-19 vaccination into its existing HIV treatment centers with the goal of offering patients and family members vaccination services, thereby rapidly expanding static vaccination site numbers in the country. Successful strategies for engaging HIV treatment centers included using existing human resources by adequately preparing HIV healthcare workers to offer vaccination and encouraging them to get vaccinated themselves, developing targeted promotional materials for persons living with HIV who are at increased risk for severe illness (*3*), and rapidly adapting and implementing similar models across the country. After this preparatory work, Zambia used the annual World AIDS Day event to launch its December Campaign to help reach African Union targets (*4*), focusing on engaging civil society leaders to endorse vaccination and using a mixed service delivery model that added community-delivered vaccination based on successful community HIV programs to existing static service delivery (Table). Some strategies were adapted from Zambia’s robust childhood vaccination program (*5*).

**Table:** Lessons learned from leveraging HIV programs to support COVID-19 vaccination, Zambia*

Pillar	Lessons
Planning and coordination	• Leverage existing in-country systems/programs/resources for COVID-19 vaccination. • Engage national, provincial, and district health bodies from the outset. • Develop district-level microplans based on standard tools that are approved at provincial and national levels. • Use joint planning by Ministry of Health, funding organizations, and provincial representatives. • Establish centralized M&E tools for national tracking of progress. • Begin with a small pilot in a few sites and rapidly iterate to improve quality, using a continuous quality-improvement approach. • Scale-up successful practices rapidly to quickly enhance effect. • Develop targets that can be implemented and achieved by lower levels (i.e., district health offices, service delivery teams).
Service delivery	• Adequately capacitate HCWs in HIV, MCH, and other clinics to deliver COVID-19 vaccines. • Invest in community mobilization and service delivery to overcome limits of a static service delivery approach and reach the greatest number of eligible persons, which means offering vaccines at public places (e.g., markets, malls, churches), chiefdoms, workplaces, congregate settings, and others. • Use existing community health services for HIV as vaccination points. • Anticipate additional human resource needs, and ensure adequate financial resources to support them.
Demand generation	• Ensure adequate HCW training in HIV and other clinics to answer patients’ and eligible family members’ questions about COVID-19 vaccines. • Encourage HCWs themselves to get vaccinated against COVID-19 by creating a safe space for unvaccinated HCWs to have their questions answered. • Engage public and private media nationally to address myths and misconceptions about COVID-19 vaccines. • Develop promotional materials that emphasize the value of COVID-19 vaccination for persons living with HIV because of the elevated risk for severe illness among members of this group. • Engage civil society (community, traditional, religious, and business leaders) to champion COVID-19 vaccination. Listen to and address their concerns about COVID-19 vaccines. • Use routine patient reminder call for upcoming visits to share information about vaccine availability in HIV clinics.
M&E	• Harmonize COVID-19 vaccine data collection in HIV and other clinics with the national COVID-19 vaccine M&E system. • Conduct frequent data analysis to inform site-level performance assessments and guide targeted quality improvement. • Generate feedback loops, particularly for poorly performing districts.
Logistics	• Push adequate vaccine supplies to each district based on their estimated target populations with the microplan. • Take inventory of health facility capacity to adequately store COVID-19 vaccines, and use existing infrastructure where possible. • Ensure that HIV clinic vaccine supply is incorporated into the wider health facility request.
Safety	• Provide AEFI training to HCWs. • Strengthen AEFI reporting system within HIV clinics.

*AEFI, adverse event following immunization; HCW, healthcare worker; MCH, maternal and child health; M&E, monitoring and evaluation.

To evaluate whether the December Campaign accelerated COVID-19 vaccination in Zambia, we conducted time-series analyses by using publicly available data (Appendix) (*1*). All participants entered in the Our World in Data dataset by February 21, 2022, for Zambia and 55 African Union member states were eligible for the analysis. We conducted 3 statistical analyses. First, in a single-group interrupted time-series analysis in Zambia only, we compared the number of persons reaching full vaccination status per day before the December 1, 2021, campaign start versus after the campaign start. Second, in a multigroup interrupted time-series analysis, we assessed whether Zambia’s acceleration in COVID-19 vaccination coverage (i.e., acceleration in the percentage of total population reaching full vaccination status per day) after the December Campaign intervention was statistically superior to 2 control groups: 2 neighboring countries with similar pre-intervention vaccination coverage trajectories and similar vaccine availability, and the average for all 55 Africa Union member states. Third, we implemented 2 sensitivity analyses for each of the above 2 analytic approaches by varying the approach to managing missing data (i.e., most recent value carried forward approach vs. interpolation approach) and comparing varied time periods for the analysis to determine the duration of December Campaign effect.

During December 2021, a total of 585,677 persons in Zambia were reached for vaccination, compared with approximately 1,071,682 million during April–November 2021. Daily COVID-19 vaccinations increased from 3,713/day before December 2021 to 17,783/day after December 1, 2021 (p<0.001) (Figure, panel A; Appendix Table 3).

**Figure:**
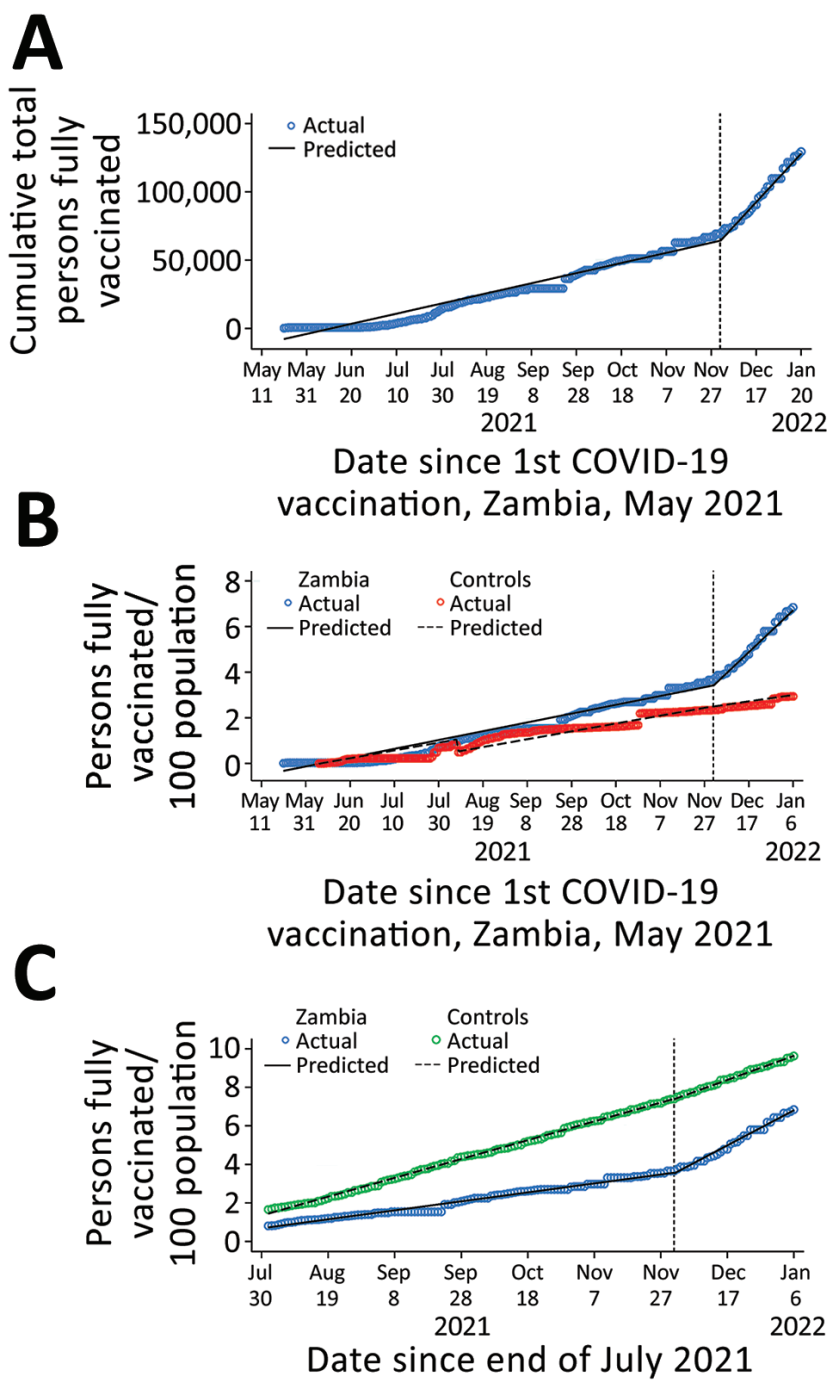
Time series of COVID-19 vaccination in Zambia, April 2021 to February 2022. A) Before and after the December Campaign. B) Compared with 2 neighboring countries with similar pre-intervention vaccination coverage trajectories and similar vaccine availability. C) Compared with the average for all 55 Africa Union member states. Prais-Winsten and Cochrane-Orcutt regression, lag(1). Vertical dashed line indicates start of Joint HIV Awareness and COVID-19 Vaccination Drive, December 1, 2021.

Compared with the average for 2 neighboring countries with similar vaccination trends before December and vaccine availability, Zambia accelerated its population COVID-19 vaccine coverage rate by an additional 2.73%/month (p<0.001) (Figure, panel B; Appendix Table 4). Compared with Africa as a whole, Zambia vaccine coverage accelerated by 1.87%/month (p<0.001) (Figure, panel C; Appendix Table 5). This accelerated vaccination in Zambia was robust to the sensitivity analysis for which we used an interpolation approach to missing data instead of the approach carrying forward the most recent available data point (Appendix Tables 3, 6). In addition, the average post-December daily vaccination rate dropped only slightly, and the average post-December percentage gain per day in a fully vaccinated population remained relatively stable, indicating a sustained effect for nearly 3 months after the December Campaign launch. If current trends were sustained, Zambia could reach its targeted 70% eligible population coverage in November 2023, ahead of other countries in Africa (August 2024) (Appendix Table 7).

For Africa to reach the 2022 Africa Union targets and adequately protect the continent from subsequent COVID-19 waves, substantially accelerated COVID-19 vaccination delivery is needed (*4*). Moreover, rapidly reaching high vaccination coverage in Africa can help reduce the risk for emergence of new variants that can rapidly spread globally (*6*,*7*). These data suggest that strong government leadership can leverage a robust HIV program, civil society, and integrated HIV donor support from the US President’s Emergency Plan for AIDS Relief and others to rapidly increase COVID-19 vaccine uptake. Zambia`s example could hasten similar adaptations in other Africa countries.

Appendix Additional methods and results for study of leveraging HIV program and civil society to accelerate COVID-19 vaccine uptake, Zambia.
