# Supplementary material for: Leveraging HIV Program and Civil Society to Accelerate COVID-19 Vaccine Uptake, Zambia
Source: Emerg Infect Dis. 2022 Dec;28(Suppl 1):S244–6. doi: 10.3201/eid2813.220743 (PMC9745228; doi:10.3201/eid2813.220743)
Supplement: Appendix — Additional methods and results for study of leveraging HIV program and civil society to accelerate COVID-19 vaccine uptake, Zambia. [file 22-0743-Techapp-s1.pdf]

Article DOI: https://doi.org/10.3201/eid2813.220743

Leveraging HIV Program and Civil Society to Accelerate COVID-19 Vaccine Uptake, Zambia

Appendix

Additional Methods

Study Design

In this interrupted time series analysis (ITSA), we analyzed publicly available data on daily COVID-19 vaccinations available from the Our World in Data (OWID) public data repository for Zambia and all 55 member states of Africa by February 21, 2022.

Data sources and participants

All participants entered into the OWID dataset by February 21, 2022, for Zambia and 55 African Union member states were eligible for the analysis. Eligibility for COVID-19 vaccinations changed over time across the 55 states of the African Union. Details of the changing vaccination policy over time can be found on the OWID Vaccination Policies webpage (https://ourworldindata.org/covid-vaccinations). A summary of the change in policy by month for three focus countries is provided in Appendix Table 1.

Intervention

The intervention of interest was the December Campaign, which started on December 1st in Zambia. Preparation for the December campaign started in August 2021, by training healthcare workers in health facilities to implement vaccinations, equipping the HIV service points at facilities and community HIV service locations, engaging community leaders through the HIV civil society network, preparing the key messages for the December campaign. Appendix 1 above describes the multiple components of the December Campaign intervention.

Outcomes

The key outcome of interest was a complete vaccination status. OWID defines people with a completed vaccination protocol as those who have received all doses prescribed by their

vaccination regimen (e.g., 2 doses for Pfizer/BioNTech, Moderna, Oxford/AstraZeneca, etc. and 1 dose for Janssen, CanSino, etc.).

Some countries also allow for alternative definitions, such as having been infected with SARS-CoV-2 in the past and having received 1 dose of a two-dose regimen. We currently ignore these alternative definitions to preserve the common definition of a complete protocol, i.e. all doses required in the vaccine regimen. This allows for optimal comparability between countries. The types of vaccines deployed in Zambia, Tanzania and Malawi are provided in Appendix Table 2.

Data management

For Zambia and two neighboring countries, data on vaccination coverage was missing on some days during the study time periods of interest. In a sensitivity analysis, we used two approaches to the missing data: (1) carrying the most recent value for complete vaccinations forward until the next available data point, and (2) using the interpolation function in STATA to cover gaps in missing data points.

Statistical analysis

We implemented the statistical analysis in three phases. First, in a single group ITSA in Zambia only, we compared the number of people reaching full vaccination status per day before the December 1, 2021, campaign start versus after the campaign start (December 1, 2021 onwards). Secondly, in a multigroup ITSA, we assessed whether Zambia`s acceleration in COVID-19 vaccination coverage (i.e., acceleration in the percentage of total population reaching full vaccination status per day) following the December Campaign intervention was statistically superior to two different control groups: (a) two neighboring countries with similar pre-intervention vaccination coverage trajectories and similar vaccine availability, and (b) the average for all 55 Africa Union member states. Thirdly, we implemented two sensitivity analyses for each of the above two analytic approaches by: (a) varying the approach to managming missing data (i.e., most recent value carried forward approach versus interpolation approach), and (b) comparing varying the time periods for the analysis to inform understanding of the duration of December campaign impact (see Appendix 8 below).

Ethical approval

Since the data are publicly available and de-identified, this analysis was considered non-research by authors and independent reviewers at the U.S. Centers for Disease Prevention and Control.

Additional Results

Single-Group ITSA

During December 2021, 585,677 persons in Zambia were reached for vaccination, compared with approximately 1,071,682 million during April to November 2021. Daily COVID-19 vaccinations rate accelerated by 14,070/day from 3,713/day before December to 17,783/day since December ($p<0.001$) (Figure 1a, Appendix 3). This statistically significant acceleration was robust to the sensitivity analysis where an interpolation approach to missing data was used instead of the approach carrying forward the most recent available data point (Appendix 3). In addition, the average post-December daily vaccination rate only dropped slightly, from 17,783/day for December 1 – January 6, 2022 to 13,911/day for December 1 – February 20, 2022, indicating a sustained impact for nearly 3 months after December Campaign intervention launch (Appendix 3).

Multi-Group ITSA

In the multi-group ITSA, compared with the two neighboring countries, Zambia accelerated its population COVID-19 vaccine coverage rate by an additional 2.73% coverage/month ($p<0.001$) (Figure 1b, Appendix 4). Compared with the average daily increase in population coverage for Africa, Zambia accelerated 1.87% coverage/month ($p<0.001$) (Figure 1c, Appendix 5). This statistically significant acceleration was robust to the sensitivity analysis where an interpolation approach to missing data was used instead of the approach carrying forward the most recent available data point (Appendix 6). In addition, the increased average post-December percentage gain per day in population fully vaccinated remained relatively stable through February 20, 2022, indicating a sustained impact for nearly 3 months after December Campaign intervention launch (Appendix 6).

Limitations

This analysis has several strengths and limitations. Strengths include the availability of daily trends in vaccination numbers and coverage, and clear timing of the intervention launch (December 1, 2021), making the interpretation of observed changes in vaccination trends as largely due to December Campaign intervention impact plausible. Limitations include the fact that the analysis relies on routinely collected program data, the analysis design is quasi-experimental which increases risk of confounding, and, given the nature of real-world program implementation, it is possible that some of the observed acceleration in vaccination coverage in Zambia occurred because of increased engagement through non-HIV service delivery points and through processes unrelated to the December Campaign.

Appendix Table 1. Vaccination policies in three focus countries for the study time period of interest*

Period	Zambia	Neighboring country 1	Neighboring country 2
March 1, 2021	No policy	No policy	No policy
April 1, 2021	No policy	All vulnerable groups	No policy
May 1, 2021	One group	Universal	No policy
June 1, 2021	All vulnerable groups	Universal	No policy
July 1, 2021	All vulnerable groups	Universal	No policy
August 1, 2021	All vulnerable groups	Universal	All vulnerable groups
Sept 1, 2021	All vulnerable groups and some others	Universal	All vulnerable groups and some others
Oct 1, 2021	All vulnerable groups and some others	Universal	Universal
Nov 1, 2021	All vulnerable groups and some others	Universal	Universal
Dec 1, 2021	All vulnerable groups and some others	Universal	Universal
Jan 1, 2022	All vulnerable groups and some others	Universal	Universal
Feb 1, 2021	All vulnerable groups and some others	Universal	Universal

*Vaccination policies are divided into 6 groups as follows: (1) No availability; (2) Availability for ONE of following: key workers/ clinically vulnerable groups / elderly groups; (3) Availability for TWO of following: key workers/ clinically vulnerable groups / elderly groups; (4) Availability for ALL of following: key workers/ clinically vulnerable groups / elderly groups; (5) Availability for all three plus partial additional availability (select broad groups/ages); (6) Universal availability.Given that the vaccination policy for all 55 member states varied significantly across countries and over time, we have not attempted to summarize the data here, but these data are available at OWID.

Appendix Table 2. Vaccinations deployed in Zambia and two neighboring countries*[2]

COVID-19 vaccines deployed in focus countries	Zambia	Neighboring country 1	Neighboring country 2
Vaccines	Johnson&Johnson, Oxford/AstraZeneca, Sinopharm/Beijing	Johnson&Johnson, Oxford/AstraZeneca	Johnson&Johnson, Pfizer/BioNTech, Sinopharm/Beijing

*Data from Our World in Data. Country-specific types of COVID-19 vaccines by February 21, 2022. (https://ourworldindata.org/covid-vaccinations).

Appendix Table 3. Single group interrupted time series analysis for Zambia, comparing pre-December with December 1, 2021-onward vaccination trends

	Pre-December Intervention (daily number of people fully vaccinated)*			Impact on daily number of complete vaccinations of December Intervention			Post-December 1 Intervention (daily number of people fully vaccinated)		
Analysis	Trend	95% CI	p-value	Additional vaccinations/day	95% CI	p-value	Trend	95% CI	p-value
Primary Analysis†									
Zambia	3,713	(2,995-4,431)	<0.001	14,070	(9,654-18,486)	<0.001	17,783	(13,640-21,927)	<0.001
Sensitivity Analysis 1‡									
Zambia	3,602	(3,219-3,986)	<0.001	12,807	(9,939-15,676)	<0.001	16,410	(13,586-19,234)	<0.001
Sensitivity Analysis 2§									
Zambia	3,610	(3,221-3,999)	<0.001	10,301	(8,532-12,070)	<0.001	13,911	(12,212-15,610)	<0.001

*Pre-December time-period: May 20, 2021 – November 30, 2021.
†Primary analysis: Missing data approach: Most recent value carried forward. Post-intervention time-period: December 1- January 6, 2022.
‡Sensitivity analysis 1: Missing data approach: Interpolation. Post-intervention time-period: December 1- January 6, 2022.
§Sensitivity analysis 2: Missing data approach: Interpolation. Post-intervention time-period: December 1- February 20, 2022.

Appendix Table 4. Multi-group interrupted time series analysis, comparing pre-December with December 1, 2021-onward vaccination trends in Zambia with two neighboring countries

Analysis	Coefficient	95% CI	p-value
Pre-December trend comparisons to assess suitability of neighboring countries as controls*			
Pre-December mean level difference in cumulative % of population vaccinated	-0.31	(-0.88-0.25)	0.279†
Pre-December difference in slope (i.e., difference in daily change in % of population vaccinated /day)	0.00	(0-0.01)	0.399†
December intervention impact on speed of daily vaccine rollout			
Acceleration in % of population vaccinated/day in Zambia compared with average for controls	0.08	(0.04-0.11)	<0.001
Absolute differences in speed of post-December vaccine rollout between Zambia and Average of controls			
Zambia`s Post-December average % of population vaccinated per day‡	0.09	(0.07-0.12)	<0.001
Neighboring Countries (i.e., Controls) Post-December 1, 2021, average % of population vaccinated per day	0.01	(0-0.03)	0.026
Absolute Difference between Post-December 1, 2021, average % of population vaccinated per day	0.08	(0.05-0.11)	<0.001

*Pre-December time period: May 20, 2021 – November 30, 2021.
†Because difference in mean baseline levels of vaccination coverage and speed or slope of percentage vaccinated per day, control group is appropriate statistically.
‡Post-December time period: December 1, 2021 – January 6, 2022.

Appendix Table 5. Results of multi-group interrupted time series analysis, comparing pre-December with December 1, 2021-onward vaccination trends in Zambia with the average for Africa

Analysis	Coefficient	95% CI	p-value
Pre-December trend comparisons to assess suitability of Africa average as a control*†			
Pre-December mean level difference in cumulative % of population vaccinated	-0.71	(-0.86--0.57)	<0.001
Pre-December difference in slope (i.e., difference in daily change in % of population vaccinated /day)	-0.03	(-0.03--0.02)	<0.001
December intervention impact on speed of daily vaccine rollout			
Acceleration in % of population vaccinated/day in Zambia compared with average for control group	0.05	(0.04-0.07)	<0.001
Absolute differences in speed of post-December vaccine rollout between Zambia and Africa average			
Zambia`s Post-December average % of population vaccinated per day‡	0.09	(0.08-0.1)	<0.001
Africa`s Post-December 1, 2021, average % of population vaccinated per day	0.06	(0.06-0.07)	<0.001
Absolute Difference between Post-December average % of population vaccinated per day between Zambia and the average for Africa	0.03	(0.02-0.04)	<0.001

*Pre-December time period: July 30, 2021 – November 30, 2021.
†Baseline levels and slopes are statistically different pre-December (i.e., Africa average is faster than Zambia), making the Africa average a less suitable control group for Zambia. However, because the pre-intervention vaccination coverage slope is higher in Africa overall, this makes it less likely that the acceleration in Zambia would be observed to be statistically significant (i.e., the approach taken is conservative). The Africa average is taken directly from Our World in Data and includes Zambia, making the comparison with Zambia more conservative (i.e., less likely to detect a significant difference).
‡Post-December time period: December 1, 2021 – January 6, 2022

Appendix Table 6. Multi-group sensitivity analysis to assess robustness of primary estimates of accelerated full vaccination coverage depending on (1) approach to missing data management and (2) time period included in the analysis after December 1, 2021 vaccination campaign launched

	Primary Analysis*			Sensitivity Analysis 1†			Sensitivity Analysis 2‡		
Robustness	Coefficient	95% CI	p-value	Coefficient	95% CI	p-value	Coefficient	95% CI	p-value
Acceleration in % of population vaccinated/day in Zambia compared with average for controls (two neighboring countries)	0.08	(0.04-0.11)	<0.001	0.06	(0.04-0.07)	<0.001	0.05	(0.04-0.06)	<0.001
Acceleration in % of population vaccinated/day in Zambia compared with average for control group (Average for Africa)	0.05	(0.04-0.07)	<0.001	0.05	(0.04-0.06)	<0.001	0.04	(0.03-0.05)	<0.001

*Primary analysis: Missing data approach: Most recent value carried forward. Post-intervention time-period: December 1- January 6, 2022.
†Sensitivity analysis 1: Missing data approach: Interpolation. Post-intervention time-period: December 1- January 6, 2022.
††Sensitivity analysis 2: Missing data approach: Interpolation. Post-intervention time-period: December 1- February 20, 2022.

Appendix Table 7. Estimated time taken to reach 70% population coverage with COVID-19 vaccination

Country	Additional % of population fully vaccinated per Day post December 1, 2021*	Additional % of population fully vaccinated per Month†	Baseline coverage population at January 6, 2022‡	Target coverage of population	Population size§	Currently vaccinated (calculated estimate)¶	Target (i.e. 70% of total population)	Numbers fully vaccinated per day at current rates#	Days needed to reach 70% target**	Months needed to reach 70% target	Date 70% target is reached
Zambia	0.09	2.73	6.84%	70%	18,384,000	1,257,466	12,868,800	16,734	694	23.13	30-Nov-23
Two neighboring countries (average)	0.01	0.42	2.94%	70%	78,864,000	2,314,658	55,204,800	11,124	4,754	158.48	12-Jan-35
Africa	0.06	1.87	9.62%	70%	1,370,700,000	131,861,340	959,490,000	856,470	966	32.21	29-Aug-24

*Taken from appendices 3 and 4 above.
†Additional % of population fully vaccinated per Month = Additional % of population fully vaccinated per day*30
‡From our world in data: https://ourworldindata.org/covid-vaccinations.
§Population **sizes** for Zambia and Africa for 2021 from https://www.statista.com/statistics/1224168/total-population-of-africa.
¶Calculated as baseline coverage population at January 6, 2022 * population size.
#Calculated as post-December additional % of population vaccinated per day multiplied by population size.
**Calculated as the total number of people that still need to be reached with full vaccination services divided by the number vaccinated per day post-December.
